# Morphology and molecular phylogeny reveal new species of *Sporocadus* (*Sporocadaceae*, *Amphisphaeriales*) in Xizang, China

**DOI:** 10.3897/mycokeys.131.187025

**Published:** 2026-04-17

**Authors:** Zixuan Li, Chengming Tian, Ning Jiang

**Affiliations:** 1 The Key Laboratory for Silviculture and Conservation of the Ministry of Education, Beijing Forestry University, Beijing 100083, China Chinese Academy of Forestry Beijing China; 2 Key Laboratory of Forest Protection of National Forestry and Grassland Administration, Ecology and Nature Conservation Institute, Chinese Academy of Forestry, Beijing 100091, China Beijing Forestry University Beijing China

**Keywords:** *
Amphisphaeriales
*, multi-gene phylogeny, new taxa, *
Rosaceae
*, systematics, taxonomic revision

## Abstract

*Sporocadus* is the type genus of *Sporocadaceae* and is phylogenetically closely related to *Seimatosporium*. Traditionally, these two genera were distinguished by the presence or absence of conidial appendages; however, this morphological character is sometimes plastic and unstable. Previous studies have also shown that internal transcribed spacer (ITS) and large subunit (LSU) sequence data are insufficient for their resolution. In this study, fungal specimens were collected from branches of two rosaceous hosts, *Prunus cerasifera* and *Rosa chinensis*, in Xizang, China. Based on morphological examination and multi-locus phylogenetic analyses of a combined ITS, LSU, *rpb2*, *tef1*, and *tub2* dataset, two new species are revealed and described herein as *Sp. bomiensis* and *Sp. prunicola***spp. nov**. Furthermore, *Seimatosporium parvum*, which possesses distinct conidial appendages, is transferred to *Sporocadus* as *Sp. parvus***comb. nov**. based on strong molecular evidence. Given the limited resolution of ITS and LSU and the instability of conidial appendages, we suggest that five-loci datasets including all accepted species are essential for accurate identification within the *Sporocadus*–*Seimatosporium* complex. This study not only expands the species diversity of *Sporocadus* in high-altitude regions but also provides new insights into the taxonomy and identification of pestalotioid fungi.

## Introduction

The family *Sporocadaceae* (*Amphisphaeriales*, *Sordariomycetes*) comprises a diverse group of fungi characterized by their acervular or pycnidial conidiomata and typically multi-septate, pigmented conidia, often bearing cellular appendages (Nag Raj 1993; Lee et al. 2006; Tanaka et al. 2011; Jiang et al. 2023; Song et al. 2023). Known as “pestalotioid-like” fungi, members of this family are widely distributed across various ecological niches as endophytes, plant pathogens, or saprobes, particularly on woody hosts (Maharachchikumbura et al. 2014; Jaklitsch et al. 2016; Jiang et al. 2018, 2021; Liu et al. 2019). The taxonomic history of *Sporocadaceae* has been complex, historically being treated under various familial names such as *Amphisphaeriaceae* or *Pestalotiopsidaceae* (Senanayake et al. 2015; Jaklitsch et al. 2016). However, recent large-scale phylogenetic revisions have consolidated these groups into a single family, *Sporocadaceae*, emphasizing the evolutionary significance of multi-locus data over plastic morphological traits (Liu et al. 2019; Peng et al. 2022; Razaghi et al. 2024).

*Sporocadus*, the type genus of *Sporocadaceae*, was initially poorly defined and lacked a designated type species (Corda 1839). It was later lectotypified with *Sp.*lichenicola (Hughes 1958). For decades, the delimitation of *Sporocadus* remained a subject of intense debate. *Sporocadus* was synonymized under a broadly defined *Seimatosporium*, primarily based on their similar conidiogenesis and septation (Sutton 1975). In contrast, Nag Raj (1993) argued for the resurrection of *Sporocadus* as a distinct genus, proposing that it should accommodate species producing 3-septate conidia that lack appendages, whereas *Seimatosporium* species typically possess appendages.

The advent of molecular phylogenetics initially added to the confusion. Early studies using only ribosomal DNA (internal transcribed spacer (ITS) and large subunit (LSU)) found that *Sporocadus*-like and *Seimatosporium*-like species often clustered together, leading some authors to maintain the synonymy (Barber et al. 2011; Tanaka et al. 2011). However, as noted by more recent studies, single-locus datasets lack sufficient resolution to differentiate these closely related genera (Liu et al. 2019; Peng et al. 2022; Razaghi et al. 2024; Yang et al. 2024). The breakthrough came with the application of multi-locus phylogenies incorporating protein-coding genes such as *rpb2*, *tef1*, and *tub2*. Liu et al. (2019) demonstrated that *Sporocadus* and *Seimatosporium* represent distinct monophyletic lineages, confirming Nag Raj’s morphological hypotheses (Nag Raj 1993). Despite this, morphological plasticity remains a challenge; some species phylogenetically nested within *Sporocadus* have been observed to produce appendages, suggesting that appendage presence/absence is a homoplastic character (Maharachchikumbura et al. 2014; Crous et al. 2018; Liu et al. 2019; Jiang et al. 2023).

Xizang (Tibet), often referred to as the “Third Pole,” is a global biodiversity hotspot characterized by unique alpine ecosystems and a high degree of endemism. Despite its vast area and diverse flora, the fungal diversity of Xizang, particularly regarding coelomycetes in *Sporocadaceae*, remains significantly understudied. In the present study, we conducted mycological surveys on rosaceous hosts in Xizang, China. Through a combination of detailed morphological examination and a comprehensive five-locus phylogenetic analysis (ITS, LSU, *rpb2*, *tef1*, and *tub2*), we aim to (1) characterize the novel species of *Sporocadus* and (2) reassess the taxonomic position of *Seimatosporium parvum* based on new molecular evidence. This work contributes to a more natural classification system for *Sporocadaceae* and enriches our knowledge of fungal diversity in the Qinghai–Xizang Plateau.

## Materials and methods

### Sample collection, isolation, and morphology

Mycological surveys were conducted in 2024 across various regions of Xizang, China. Specimens were obtained from branch cankers of *Prunus cerasifera* and dead branches of *Rosa chinensis*. Collected branches were cut into 15 cm segments and transported to the laboratory in paper envelopes.

Pure cultures were obtained using the single-spore isolation method. Spore masses were suspended in sterile distilled water to create a spore suspension, which was then streaked onto the surface of potato dextrose agar (PDA) plates. After incubation at 25 °C for 24 hours, germinating spores were identified under a stereomicroscope and individually transferred to PDA and malt extract agar (MEA) plates. The resulting isolates were incubated at 25 °C in the dark to observe colony morphology and growth rates. Type specimens were deposited in the Herbarium of the Chinese Academy of Forestry (**CAF**), and ex-type living cultures were stored in the China Forestry Culture Collection Center (**CFCC**).

Morphological features were initially examined on the natural substrates using a Zeiss Discovery V8 stereomicroscope (Oberkochen, Germany). Detailed structures of the ascomata, asci, ascospores, conidiomata, conidiophores, conidiogenous cells, and conidia were further scrutinized and photographed using an Olympus BX51 microscope (Tokyo, Japan). Quantitative data were obtained by measuring at least 50 representative spores, and measurements are presented as (min–) (mean–SD)–(mean + SD) (–max). Colony characteristics on PDA and MEA, including texture, color, and pigment production, were recorded after 14 days of incubation.

### Phylogenetic analyses

Genomic DNA was extracted from fungal mycelia harvested from 14-day-old cultures on PDA using the CTAB method. Five gene loci were amplified and sequenced, including the internal transcribed spacer (ITS), the large subunit of the ribosomal DNA (LSU), the RNA polymerase II second largest subunit (*rpb2*), the translation elongation factor 1-alpha (*tef1*), and the beta-tubulin (*tub2*). The primers used for PCR were ITS1/ITS4 for ITS (White et al. 1990), LR0R/LR5 for LSU (Vilgalys and Hester 1990), RPB2-5F/RPB2-7cR for *rpb2* (Liu et al. 1999), EF1-728F/EF1-986R for *tef1* (Carbone and Kohn 1999), and Bt2a/Bt2b for *tub2* (Glass and Donaldson 1995). PCR reactions were performed in a total volume of 25 μL containing 12.5 μL of 2× Master Mix, 1 μL of each primer (10 μM), 1 μL of DNA template, and 9.5 μL of ddH_2_O. The amplification programs followed the thermal cycling conditions described by Liu et al. (2019). PCR products were visualized via 1% agarose gel electrophoresis and sequenced by Ruibiotech Co., Ltd. (Beijing, China).

The quality of raw sequences was assessed, and consensus sequences were assembled using MEGA v.7 (Kumar et al. 2016). Novel sequences generated in this study were supplemented with reference sequences retrieved from GenBank (Table 1). Sequence alignments for each locus were performed using MAFFT v.7.110 and manually adjusted where necessary (Katoh and Standley 2013). The five single-locus alignments were concatenated using SequenceMatrix v.1.7.8.

**Table 1.** GenBank accession numbers used in the phylogenetic analyses.

**Species**	**Strain**	**GenBank accession numbers**					**References**
		**ITS**	**LSU**	***rpb2***	***tef1***	***tub2***	
*Allelochaeta acuta*	CBS 144168	MH822973	MH823023	MH823071	MH823113	MH823160	Crous et al. 2018
*Allelochaeta falcata*	CBS 131117*	MH553999	MH554217	MH554907	MH554426	MH554668	Liu et al. 2019
*Allelochaeta obliquae*	CBS 144182*	MH554105	MH554315	MH555018	MH554539	MH554778	Liu et al. 2019
*Bartalinia bella*	CBS 464.61*	MH554051	MH554264	MH554964	MH554486	MH554727	Liu et al. 2019
*Bartalinia pini*	CBS 143891*	MH554125	MH554330	MH555033	MH554559	MH554797	Liu et al. 2019
*Broomella vitalbae*	HPC 1154	MH554173	MH554367	MH555069	MH554608	MH554846	Liu et al. 2019
*Cavernicola guangxiensis*	LC15867	OR247933	OR247974	OR792176	OR361513	OR380985	Razaghi et al. 2024
*Ciliochorella phanericola*	MFLUCC 14-0984*	KX789680	KX789681	NA	NA	KX789682	Liu et al. 2019
*Diploceras hypericinum*	CBS 143885*	MH554108	MH554316	MH555019	MH554542	MH554781	Liu et al. 2019
*Disaeta arbuti*	CBS 143903*	MH554148	MH554346	MH555050	MH554583	MH554821	Liu et al. 2019
*Discosia artocreas*	CBS 124848*	MH553994	MH554213	MH554903	MH554420	MH554662	Liu et al. 2019
*Discosia ascidiata*	LC1107	OR247912	OR247956	OR792160	OR361484	OR380987	Razaghi et al. 2024
*Discosia castaneae*	LC12213	OR247913	OR247957	OR792161	OR361485	OR380988	Razaghi et al. 2024
*Distononappendiculata banksiae*	CBS 131308*	JQ044422	JQ044442	MH554909	MH554428	MH554670	Crous et al. 2011
*Distononappendiculata casuarinae*	CBS 143884*	MH554093	MH554303	MH555007	MH554527	MH554766	Liu et al. 2019
*Distononappendiculata verrucata*	CBS 144032*	MH554163	MH554359	MH555062	MH554598	MH554836	Liu et al. 2019
*Diversimediispora humicola*	CBS 302.86*	MH554028	MH554247	MH554941	MH554463	MH554705	Liu et al. 2019
*Heterotruncatella longissima*	CBS 143910*	MH554165	MH554361	MH555064	MH554600	MH554838	Liu et al. 2019
*Heterotruncatella proteicola*	CBS 144020*	MH554077	MH554288	MH554989	MH554512	MH554751	Liu et al. 2019
*Heterotruncatella restionacearum*	CBS 118150	DQ278914	MH554203	MH554889	MH554407	MH554649	Lee et al. 2006
*Hyalotiella transvalensis*	CBS 303.65*	MH554029	MH554248	MH554942	MH554464	MH554706	Liu et al. 2019
*Hymenopleella austroafricana*	CBS 143886*	MH554115	MH554320	MH555023	MH554549	MH554788	Liu et al. 2019
*Hymenopleella hippophaeicola*	CBS 140410*	NR_154078	MH554224	MH554919	MH554436	MH554678	Jaklitsch et al. 2016; Liu et al. 2019
*Hymenopleella polyseptata*	CBS 143887*	MH554116	MH554321	MH555024	MH554550	MH554789	Liu et al. 2019
*Immersidiscosia eucalypti*	KUNCC 23-15527	PP584710	PP584807	NA	NA	NA	Dissanayake et al. 2024
*Immersidiscosia eucalypti*	MFLU 16-1372	MF173609	MF173608	NA	NA	NA	Dissanayake et al. 2024
*Lepteutypa fuckelii*	CBS 140409*	NR_154123	KT949902	MH554918	MH554435	MH554677	Liu et al. 2019
*Monochaetia monochaeta*	CBS 199.82*	MH554018	MH554238	MH554931	MH554452	MH554694	Liu et al. 2019
*Monochaetia quercus*	CBS 144034*	MH554171	MH554365	MH555068	MH554606	MH554844	Liu et al. 2019
*Morinia acaciae*	CBS 137994*	MH554002	MH554221	MH554914	MH554431	MH554673	Crous et al. 2014
*Morinia crini*	CBS 143888*	MH554118	MH554323	MH555026	MH554552	MH554791	Liu et al. 2019
*Neopestalotiopsis rosae*	CBS 101057*	KM199359	KM116245	MH554850	KM199523	KM199429	Maharachchikumbura et al. 2014
*Neopestalotiopsis surinamensis*	CBS 450.74*	KM199351	KM116258	MH554962	KM199518	KM199465	Maharachchikumbura et al. 2014
*Nonappendiculata quercina*	CBS 116061*	MH553982	MH554199	MH554882	MH554400	MH554641	Liu et al. 2019
*Parabartalinia lateralis*	CBS 399.71*	MH554043	MH554256	MH554954	MH554478	MH554719	Liu et al. 2019
*Pestalotiopsis australasiae*	CBS 114126*	KM199297	KM116218	MH554867	KM199499	KM199409	Maharachchikumbura et al. 2014
*Pestalotiopsis australis*	CBS 114193*	KM199332	KM116197	MH554875	KM199475	KM199383	Maharachchikumbura et al. 2014
*Pestalotiopsis grevilleae*	CBS 114127*	KM199300	KM116212	MH554868	KM199504	KM199407	Maharachchikumbura et al. 2014
*Phlogicylindrium uniforme*	CBS 131312	JQ044426	JQ044445	NA	NA	MH704634	Crous et al. 2015
*Pseudopestalotiopsis cocos*	CBS 272.29*	KM199378	KM116276	MH554938	KM199553	KM199467	Maharachchikumbura et al. 2014
*Pseudopestalotiopsis elaeidis*	CBS 413.62*	MH554044	MH554257	MH554955	MH554479	MH554720	Liu et al. 2019
*Pseudopestalotiopsis indica*	CBS 459.78*	KM199381	MH554263	MH554963	KM199560	KM199470	Maharachchikumbura et al. 2014
*Pseudosarcostroma osyridicola*	CBS 103.76*	MH553954	MH554177	MH554851	MH554372	MH554613	Liu et al. 2019
*Robillarda australiana*	CBS 143882	MH554091	MH554301	MH555005	MH554525	MH554764	Liu et al. 2019
*Robillarda roystoneae*	CBS 115445*	KR873254	KR873282	MH554880	KR873310	KR873317	Crous et al. 2015
*Robillarda sessilis*	CBS 114312*	KR873256	KR873284	MH554877	KR873312	KR873319	Crous et al. 2015; Liu et al. 2019
*Sarcostroma grevilleae*	ICMP 10981	AF405304	AF382372	NA	NA	NA	Liu et al. 2019
*Sarcostroma leucospermi*	CBS 111290*	MH554081	MH554292	MH554993	MH554516	MH554755	Liu et al. 2019
*Sarcostroma paragrevilleae*	CBS 114142*	MH553974	MH554193	MH554871	MH554392	MH554633	Liu et al. 2019
*Sarcostroma proteae*	CBS 113610*	MH553968	MH554187	MH554862	MH554386	MH554627	Liu et al. 2019
*Sarcostroma restionis*	CBS 118154*	DQ278922	DQ278924	MH554891	MH554409	MH554651	Lee et al. 2006
*Seimatosporium botan*	NBRC 104200*	AB594799	AB593731	NA	NA	LC047770	Norphanphoun et al. 2015
*Seimatosporium centrale*	CFCC 55166*	OK560629	OK560399	ON055447	OM986918	OM301641	Peng et al. 2022
*Seimatosporium centrale*	CFCC 55169	OK560632	OK560402	ON055450	OM986921	OM301644	Peng et al. 2022
*Seimatosporium chinense*	CFCC 70988*	PQ279535	PQ279531	PQ283812	PQ287323	PQ287325	Yang et al. 2024
*Seimatosporium chinense*	N001A	PQ279536	PQ279532	PQ283813	PQ287324	PQ287326	Yang et al. 2024
*Seimatosporium cyprium*	CBS 149019*	ON680684	ON705769	NA	ON863790	ON695856	Kanetis et al. 2022
*Seimatosporium cyprium*	L112	ON695889	ON692404	NA	ON863791	ON695848	Kanetis et al. 2022
*Seimatosporium discosioides*	NBRC 10420	AB594800	AB593732	NA	NA	LC047771	Norphanphoun et al. 2015
*Seimatosporium germanicum*	CBS 437.87*	MH554047	MH554259	MH554957	MH554482	MH554723	Liu et al. 2019
*Seimatosporium gracile*	CFCC 55167*	OK560638	OK560408	ON055456	OM986927	OM301650	Peng et al. 2022
*Seimatosporium luteosporum*	CBS 142599*	KY706284	KY706309	NA	KY706334	KY706259	Lawrence et al. 2018
*Seimatosporium marivanicum*	CBS 143781*	MW361952	MW361960	NA	MW375358	MW375352	Moghadam et al. 2022
*Seimatosporium marivanicum*	CBS 143780	MW361951	MW361959	NA	MW375357	MW375351	Moghadam et al. 2022
*Seimatosporium nonappendiculatum*	CFCC 55168*	OK560657	OK560427	ON055475	OM986946	OM301669	Peng et al. 2022
*Seimatosporium physocarpi*	CBS 139968*	KT198722	KT198723	MH554917	MH554434	MH554676	Norphanphoun et al. 2015
*Seimatosporium physocarpi*	CBS 789.68	MH554066	MH554278	MH554979	MH554502	MH554742	Liu et al. 2019
*Seimatosporium pistaciae*	CPC 24455*	KP004463	KP004491	MH554915	MH554432	MH554674	Liu et al. 2019
*Seimatosporium pistaciae*	CPC 24457	MH554126	MH554331	MH555035	MH554561	MH554799	Liu et al. 2019
*Seimatosporium rosae*	CBS 139823*	KT198726	KT198727	LT853153	LT853203	LT853253	Norphanphoun et al. 2015
*Seimatosporium soli*	CBS 941.69*	MH554071	MH554282	MH554983	MH554507	NA	Liu et al. 2019
*Seimatosporium tibetense*	CGMCC 3.23503*	OR247936	OR247954	OR380975	OR361511	OR381084	Razaghi et al. 2024
*Seimatosporium tibetense*	LC15857	OR247937	OR247955	OR380976	OR361512	OR381085	Razaghi et al. 2024
*Seimatosporium tostum*	NBRC 32626	AB594795	AB593727	NA	NA	NA	Tanaka et al. 2011; Rossman et al. 2016
*Seimatosporium vitifusiforme*	CBS 142600*	KY706296	KY706321	NA	KY706346	KY706271	Lawrence et al. 2018
*Seimatosporium vitis*	MFLUCC 14-0051*	KR920363	KR920362	NA	NA	NA	Senanayake et al. 2015
*Seimatosporium *	CBS 123004*	MH553992	MH554211	MH554901	MH554418	MH554660	Liu et al. 2019
*Seimatosporium vitis-viniferae*	CBS 116499	MH553984	MH554201	MH554884	MH554402	MH554643	Liu et al. 2019
*Seiridium marginatum*	CBS 140403*	NR_156602	MH554223	LT853149	LT853199	LT853249	Bonthond et al. 2018
*Seiridium neocupressi*	CBS 142625*	LT853079	MH554329	LT853127	LT853176	LT853226	Bonthond et al. 2018; Liu et al. 2019
*Seiridium rhododendri*	LC15851	OR247930	OR247951	OR792180	OR361508	OR381089	Razaghi et al. 2024
*Sporocadus biseptatus*	CBS 110324*	MH553956	MH554179	MH554853	MH554374	MH554615	Liu et al. 2019
***Sporocadus bomiensis* sp. nov**.	CFCC 71463*	**PX974052**	**PX974056**	**PX981916**	**PX981920**	**PX981924**	**This study**
***Sporocadus bomiensis* sp. nov**.	CFCC 71588*	**PX974053**	**PX974057**	**PX981917**	**PX981921**	**PX981925**	**This study**
*Sporocadus brevis*	CFCC 55170*	OK655780	OK560371	OL742155	OL814537	OM401659	Peng et al. 2022
*Sporocadus brevis*	ROC 092	OK655781	OK560372	OL742156	OL814538	OM401660	Peng et al. 2022
*Sporocadus cavernicola*	CGMCC 3.23173*	OR357758	OR247948	OR380981	OR361499	OR381096	Razaghi et al. 2024
*Sporocadus cavernicola*	LC15863	OR357759	OR247949	OR380982	OR361500	OR381097	Razaghi et al. 2024
*Sporocadus corni*	MFLUCC 14-0467*	KT162918	KR559739	NA	NA	NA	Senanayake et al. 2015
*Sporocadus cornicola*	MFLUCC 14-0448*	KU974967	NA	NA	NA	NA	Wijayawardene et al. 2016
*Sporocadus cornicola*	CBS 143889	MH554121	MH554326	MH555029	MH554555	MH554794	Liu et al. 2019
*Sporocadus cotini*	CBS 139966*	MH554003	MH554222	MH554916	MH554433	MH554675	Liu et al. 2019
*Sporocadus hyperici*	CGMCC 3.23174*	OR357754	OR247944	OR380977	OR361501	OR381092	Razaghi et al. 2024
*Sporocadus hyperici*	LC15844	OR357755	OR247945	OR380978	OR361502	OR381093	Razaghi et al. 2024
*Sporocadus incanus*	CBS 123003*	MH553991	MH554210	MH554900	MH554417	MH554659	Liu et al. 2019
*Sporocadus incarnatus*	CBS 149301*	OP038025	OP076913	OP095241	NA	OP079858	Travadon et al. 2022
*Sporocadus italicum*	MFLUCC 14-1196*	MF614831	MF614829	NA	NA	NA	Hyde et al. 2017
*Sporocadus kurdistanicus*	IRAN 2356C*	MW361950	MW361958	NA	MW375356	MW375350	Moghadam et al. 2022
*Sporocadus kurdistanicus*	IRAN 2355C	MW361949	NA	NA	NA	NA	Moghadam et al. 2022
*Sporocadus kurdistanicus*	IRAN 2354C	MW361948	MW361957	NA	MW375355	MW375349	Moghadam et al. 2022
*Sporocadus kurdistanicus*	IRAN 2313C	MW361947	MW361956	NA	MW375354	MW375348	Moghadam et al. 2022
*Sporocadus lichenicola*	CBS 354.90*	MH554035	MH554252	MH554948	MH554470	MH554711	Liu et al. 2019
*Sporocadus lichenicola*	NBRC 32625	MH883643	MH883646	MH883647	MH883644	MH883645	Liu et al. 2019
*Sporocadus mali*	CBS 446.70*	MH554049	MH554261	MH554960	MH554484	MH554725	Liu et al. 2019
*Sporocadus microcyclus*	CBS 424.95*	MH554045	MH554258	MH554956	MH554480	MH554721	Liu et al. 2019
*Sporocadus microcyclus*	CBS 887.68	MH554068	MH554280	MH554981	MH554504	MH554744	Liu et al. 2019
*Sporocadus multiseptatus*	CBS 143899*	MH554141	MH554343	MH555047	MH554576	MH554814	Liu et al. 2019
*Sporocadus parvus* comb. nov. (syn. *Seimatosporium parvum*)	CFCC 55164*	OK560647	OK560417	ON055465	OM986936	OM301659	Peng et al. 2022
*Sporocadus parvus* comb. nov. (syn. *Seimatosporium parvum*)	CFCC 55165	OK560653	OK560423	ON055471	OM986942	OM301665	Peng et al. 2022
***Sporocadus prunicola* sp. nov**.	CFCC 71042*	**PX974054**	**PX974058**	**PX981918**	**PX981922**	**PX981926**	**This study**
***Sporocadus prunicola* sp. nov**.	CFCC 71288*	**PX974055**	**PX974059**	**PX981919**	**PX981923**	**PX981927**	**This study**
*Sporocadus pseudocorni*	MFLUCC 13-0529*	NA	KU359033	NA	NA	NA	Wijayawardene et al. 2016
*Sporocadus rosarum*	MFLU 14-0468	NA	KU359035	NA	NA	NA	Li et al. 2016
*Sporocadus rosarum*	MFLUCC 15-0563*	MG828960	MG829071	NA	NA	NA	Liu et al. 2019
*Sporocadus rosarum*	MFLUCC 14-0466	KT284775	KT281912	NA	NA	NA	Ariyawansa et al. 2015
*Sporocadus rosarum*	CBS 113832	MH553970	MH554189	MH554864	MH554388	MH554629	Liu et al. 2019
*Sporocadus rosigena*	ICMP 7003	NA	AF382374	NA	NA	NA	Jeewon et al. 2002
*Sporocadus rosigena*	CBS 466.96	MH554052	MH554265	MH554965	MH554487	MH554728	Liu et al. 2019
*Sporocadus rosigena*	CBS 129166	MH553996	MH554215	MH554905	MH554423	MH554665	Liu et al. 2019
*Sporocadus rosigena*	CBS 116498	MH553983	MH554200	MH554883	MH554401	MH554642	Liu et al. 2019
*Sporocadus rotundatus*	CBS 616.83*	MH554060	MH554273	MH554974	MH554496	MH554737	Liu et al. 2019
*Sporocadus sorbi*	MFLUCC 14-0469*	KT284774	KT281911	NA	NA	NA	Ariyawansa et al. 2015
*Sporocadus sorbi*	CBS 160.25	MH554008	MH554229	MH554924	MH554442	MH554684	Liu et al. 2019
*Sporocadus spiniger*	ROC 119*	OK655791	OK560382	OL742166	OL814548	OM401670	Peng et al. 2022
*Sporocadus spiniger*	ROC 120	OK655792	OK560383	OL742167	OL814549	OM401671	Peng et al. 2022
*Sporocadus tibetensis*	CGMCC 3.23172*	OR357756	OR247946	OR380979	OR361503	OR381094	Razaghi et al. 2024
*Sporocadus tibetensis*	LC15858	OR357757	OR247947	OR380980	OR361504	OR381095	Razaghi et al. 2024
*Sporocadus trimorphus*	CBS 114203*	MH553977	MH554196	MH554876	MH554395	MH554636	Liu et al. 2019
*Strickeria kochii*	CBS 140411*	NR_154423	NG_064298	MH554920	MH554437	MH554679	Jaklitsch et al. 2016; Liu et al. 2019
*Synnemapestaloides juniperi*	CBS 477.77*	MH554053	MH554266	MH554966	MH554488	MH554729	Liu et al. 2019
*Truncatella angustata*	CBS 144025 NT	MH554112	MH554318	MH555021	MH554546	MH554785	Liu et al. 2019
*Truncatella angustata*	CBS 338.32	MH554033	MH554250	MH554945	MH554467	MH554709	Liu et al. 2019
*Xenoseimatosporium quercinum*	MFLUCC 14-1198*	NR_155804	NG_059681	NA	NA	NA	Liu et al. 2019
*Xenoseimatosporium quercinum*	CBS 129171	MH553997	MH554216	MH554906	MH554424	MH554666	Liu et al. 2019

**Note**. “NA” indicates unavailable sequences; sequences produced in the current study are in bold, and * means ex-type strains.

Phylogenetic trees were constructed using maximum likelihood (ML) and Bayesian inference (BI) analyses. ML analysis was performed using RAxML-HPC v.8.2.12 on the CIPRES Science Gateway, employing the GTRGAMMA model with 1,000 bootstrap replicates (Miller et al. 2012). BI analysis was conducted in MrBayes v.3.2.6 (Ronquist et al. 2012). The best-fit evolutionary models for each partition were determined by ModelFinder under the Akaike Information Criterion (AIC). Six Markov Chain Monte Carlo (MCMC) chains were run for 5,000,000 generations, sampling every 1,000^th^ generation. The first 25% of trees were discarded as burn-in, and the remaining trees were used to calculate posterior probabilities (PP). Phylograms were visualized in FigTree v.1.4.2 and edited in Adobe Illustrator (Rambaut 2014).

To further confirm the distinctiveness of the new species, the Pairwise Homoplasy Index (PHI) test was performed using SplitsTree v.4.16.1 (Huson and Bryant 2006). A PHI test (Фw) value above 0.05 (*P* > 0.05) indicates no significant recombination between the hypothesized species and its closest relatives. The phylogenetic network based on the concatenated five-locus dataset was constructed using the NeighborNet algorithm to visualize the relationships and potential recombination events.

## Results

### Phylogenetic analyses

**Figure 1.**
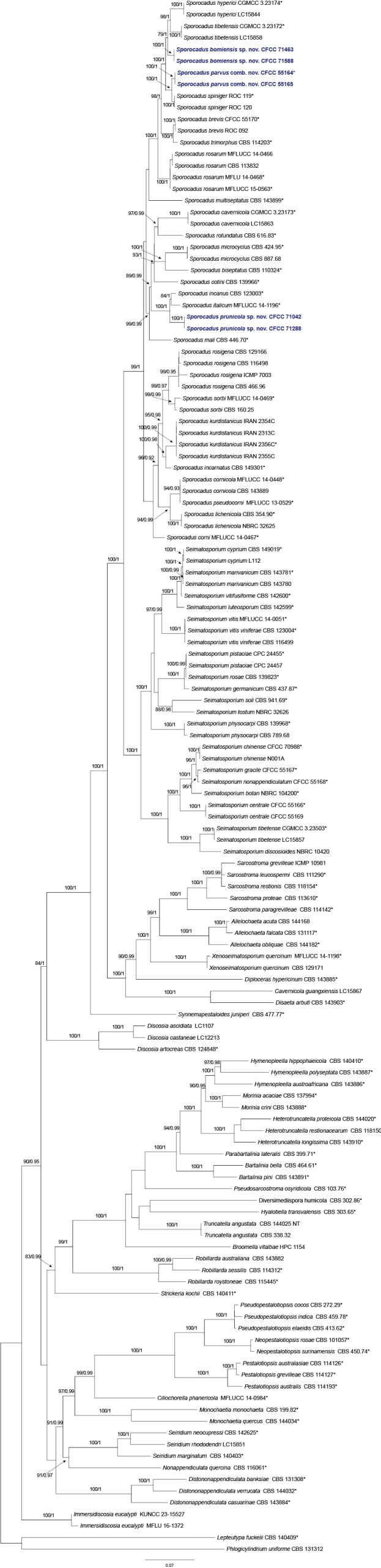
Maximum likelihood tree of *Sporocadaceae* generated from combined ITS, LSU, *rpb2*, *tef1*, and *tub2* sequence data. Bootstrap support values ≥ 50% and Bayesian posterior probabilities ≥ 0.90 are demonstrated at the branches. Isolates from the present study are indicated in blue, and * indicates ex-type strains.

The combined five-gene dataset (ITS, LSU, *rpb2*, *tef1*, and *tub2*) for the phylogenetic analysis of *Sporocadus* and its closely related genera within *Sporocadaceae* consisted of 134 strains. *Lepteutypa fuckelii* (CBS 140409) and *Phlogicylindrium uniforme* (CBS 131312) were selected as outgroup taxa. The final alignment comprised 3324 characters (including gaps), with the following partition lengths: ITS (1–513 bp), LSU (514–1292 bp), *rpb2* (1293–2199 bp), *tef1* (2200–2629 bp), and *tub2* (2630–3324 bp). The best-scoring RAxML tree (Fig. 1) had a final likelihood value of –62787.09. For the Bayesian analysis, ModelFinder selected the following best-fit models: SYM+I+G4 for ITS, TIM2+F+R3 for LSU, TIM3+F+R4 for *rpb2*, TIM3+F+R4 for *tef1*, and HKY+F+R4 for *tub2*.

Phylogenetic analyses using both maximum likelihood (ML) and Bayesian inference (BI) yielded nearly identical topologies. The multi-locus tree resolved *Sporocadus* as a well-supported monophyletic lineage. Our two new species and the new combination were strategically positioned within the *Sporocadus* clade. *Sporocadus bomiensis* sp. nov. (CFCC 71463 and CFCC 71588) formed a distinct and highly supported monophyletic lineage (MLBS = 100%, BIPP = 1.0). It clustered as a sister group to the clade containing *Sp. tibetensis* and *Sp. hyperici*, with statistical support (MLBS = 79%, BIPP = 1.0). *Sporocadus parvus* comb. nov. (CFCC 55164 and CFCC 55165) formed a robust clade (MLBS = 100%, BIPP = 1.0) that is phylogenetically closely related to *Sp. spiniger*. *Sporocadus prunicola* sp. nov. (CFCC 71042 and CFCC 71288) clustered independently (MLBS = 100%, BIPP = 1.0) and showed a close relationship to *Sp. incanus* and *Sp. italicum*, forming a larger subclade with strong branch support (MLBS = 100%, BIPP = 1.0). The results of the multi-locus analysis provided high resolution for delimiting these novel taxa, distinguishing them clearly from other previously known species.

To further validate the delineation of the newly proposed species and assess the potential for genetic recombination with their closest phylogenetic relatives, a Pairwise Homoplasy Index (PHI) analysis was conducted using a five-gene concatenated dataset. Two distinct clusters were selected for analysis based on the multi-locus phylogeny: Clade A included the new species *Sp. bomiensis* and its closely related taxa, *Sp. hyperici* and *Sp. tibetensis*. The PHI test resulted in a *P*-value of 1.0, indicating no significant evidence of genetic recombination among these species. Clade B comprised the new species *Sp. prunicola* and seven allied taxa: *Sp. cavernicola*, *Sp. rotundatus*, *Sp. microcyclus*, *Sp. biseptatus*, *Sp. cotini*, *Sp. incanus*, and *Sp. italicum*. The analysis yielded a *P*-value of 1.0, similarly suggesting an absence of significant recombination events within this group.

The split graphs generated from the NeighborNet algorithm visually confirm these results, showing clear reticulation-free relationships between the novel taxa and their congeners. Collectively, the PHI test results (*P* = 1.0) provide robust statistical support for recognizing *Sp. bomiensis* and *Sp. prunicola* as distinct and stable taxonomic entities.

### Taxonomy

#### Sporocadus bomiensis

Taxon classificationFungiAmphisphaerialesSporocadaceae

C.M. Tian & Ning Jiang sp. nov.

2BCB4191-E8E7-53C1-A837-3CC98CA191A3

862199

Fig. 3

##### Etymology.

Named after the collection site of the type specimen, Bomi County.

##### Description.

Saprobic on the bark of *Rosa chinensis*. **Teleomorph: *Ascomata*** solitary or gregarious, semi-immersed, subglobose, dark brown to black, 250–550 μm diam, 150–350 μm high. ***Peridium*** composed of textura angularis, outer region composed of relatively small, pigmented, thick-walled cells, inner with thin-walled hyaline cells, 20–50 μm wide. ***Asci*** 8-spored, unitunicate, clavate to broadly cylindrical, short pedicel or sessile, rounded at the apex, 76.5–111 × 7.5–10.5 μm. ***Ascospores*** ellipsoidal-fusiform, hyaline, smooth-walled, 3-septate, narrowly fusoid with rounded ends, (10.5–)13–15.5(–17) × (3.5–)5–6.5(–7.5) μm (x̄ = 14.3 × 5.8 μm, *n* = 50), L/W = 2.2–2.8, median cells 3.2–3.9 μm long, terminal cells 3.3–4.3 μm long.

##### Culture characteristics.

Colonies on PDA flat, spreading, with moderate aerial mycelium and undulating margin, vinaceous buff, fast growing, reaching 90 mm diam after 2 wk at 25 °C, sterile. Colonies on MEA flat, spreading, with moderate aerial mycelium and lobate margin, white, slow growing, reaching 50 mm diam after 2 wk at 25 °C, sterile.

##### Materials examined.

China • Xizang Autonomous Region (Tibet), Linzhi City, Bomi County, 29°52'18"N, 95°44'7"E, 2192 m asl, from dead branches of *Rosa chinensis*, 24 Oct. 2024, *Ning Jiang, Min Liu, Jieting Li & Yi Li* (**holotype** CAF800148, ex-holotype cultures CFCC 71463 and CFCC 71588).

##### Notes.

*Sporocadus bomiensis*, collected from *Rosa chinensis* in Xizang, is phylogenetically closely related to *Sp. hyperici* (from *Hypericum* sp.) and *Sp. tibetensis* (from an unknown host). While *Sp. bomiensis* is currently known only by its teleomorph, both *Sp. hyperici* and *Sp. tibetensis* have only been described in their anamorphic states (Razaghi et al. 2024). Morphological comparison between these taxa is thus limited; however, *Sp. bomiensis* can be clearly distinguished by significant nucleotide differences in the multi-locus alignment. Specifically, it differs from *Sp. hyperici* by 2/512 bp in ITS, 1/815 bp in LSU, 19/832 bp in *rpb2*, 55/312 bp in *tef1*, and 61/716 bp in *tub2*. From *Sp. tibetensis*, it differs by 3/513 bp in ITS, 3/815 bp in LSU, 17/832 bp in *rpb2*, 50/310 bp in *tef1*, and 61/716 bp in *tub2*.

#### Sporocadus parvus

Taxon classificationFungiAmphisphaerialesSporocadaceae

(C. Peng & C.M. Tian) C.M. Tian & Ning Jiang comb. nov.

42C4C124-1628-58F7-9410-F690F9002D8E

862200

Seimatosporium parvum C. Peng & C.M. Tian, Persoonia 49: 240 (2022). Basionym.

##### Description.

See Peng et al. (2022).

##### Notes.

*Seimatosporium parvum* was originally characterized by having distinct appendages at both the apical and basal ends of the conidia, a feature that led to its initial placement within the genus *Seimatosporium* (Peng et al. 2022). However, our current multi-locus phylogenetic analysis based on five gene loci (ITS, LSU, *rpb2*, *tef1*, and *tub2*) clearly places this species within the *Sporocadus* lineage with high statistical support (MLBS = 100%, BIPP = 1.0) (Fig. 1). Recent taxonomic revisions of *Sporocadaceae* have demonstrated that the presence or absence of conidial appendages is not a strictly conserved trait at the generic level within the *Sporocadus*–*Seimatosporium* complex (Liu et al. 2019). Consequently, based on the robust molecular evidence presented here, this fungus is formally transferred to *Sporocadus* as *Sp. parvus*.

#### Sporocadus prunicola

Taxon classificationFungiAmphisphaerialesSporocadaceae

C.M. Tian & Ning Jiang sp. nov.

0A41C98D-A4A0-51B3-88A1-DE23001A3236

862201

Fig. 4

##### Etymology.

Named after the host genus *Prunus* and and “-*cola*” = “inhabiting”.

##### Description.

Associated with branch canker disease of *Prunus cerasifera*. **Teleomorph**. Undetermined. **Anamorph: *Conidiomata*** acervular, solitary or gregarious, semi-immersed, subglobose, superficial to semi-immersed, pale luteous, 98–431 μm diam, 153–289 μm high. ***Conidiophores*** septate, branched at base, hyaline, smooth. ***Conidiogenous cells*** discrete, filiform, hyaline, smooth, 18–45 × 1–2 μm. ***Conidia*** straight or slightly curved, subcylindrical, with round ends, brown, 3-septate, septa darker than the rest of cell, wall smooth and slightly constricted at the septa, (10.5–)12–15(–16.5) × (3–)3.5–4.5(–7) μm (x̄ = 13.4 × 4.2 μm, *n* = 50), L/W = 2.8–3.7, lacking appendages; ***basal cell*** obconic with round base, pale brown, paler than other cells, thick-walled, 3–3.5 μm long; ***median cells*** doliiform, brown, 2.5–4 μm long; ***apical cell*** conic with a wide round apex, brown, 2.5–4 μm long.

##### Culture characteristics.

Colonies on PDA flat, spreading, with abundant flocculent aerial mycelium and entire margin, isabelline, fast growing, reaching 90 mm diam after 2 wk at 25 °C, sterile. Colonies on MEA flat, spreading, with moderate aerial mycelium and feathery margin, white to pale gray, fast growing, reaching 90 mm diam after 2 wk at 25 °C, sterile.

##### Materials examined.

China • Xizang Autonomous Region (Tibet), Linzhi City, Bayi District, Xizang Agriculture and Animal Husbandry University, 29°39'59"N, 94°20'20"E, 3007 m asl, from cankered branches of *Prunus cerasifera*, 8 Jul. 2024, *Ning Jiang, Jieting Li*, *Yi Li & Ji Qiang* (**holotype** CAF800149, ex-holotype cultures CFCC 71042 and CFCC 71288).

##### Notes.

**Figure 2.**
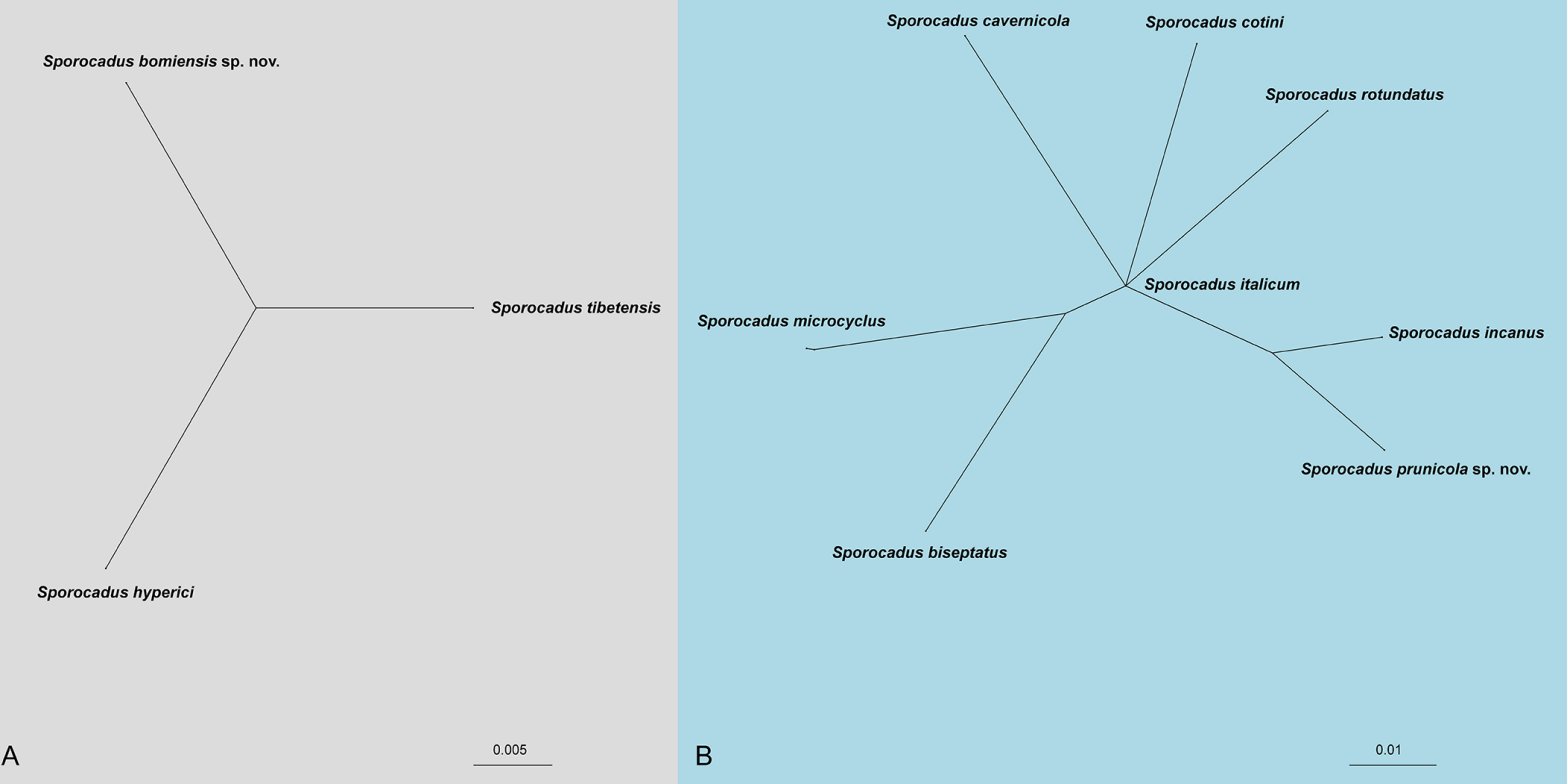
Phylogenetic network from concatenated data (ITS, LSU, *rpb2*, *tef1*, and *tub2*) representing the structure of the *Sporocadus* species, based on LogDet transformation and the NeighborNet algorithm, inferred by SplitsTree. The scale bar represents the expected number of substitutions per nucleotide position. **A**. *P* = 1.0; **B**. *P* = 1.0.

*Sporocadus prunicola*, isolated from *Prunus cerasifera* in Xizang, is phylogenetically closely related to *Sp. incanus* (from *Prunus dulcis* in Spain) and *Sp. italicum* (from *Crataegus* sp. in Italy) (Fig. 1). Morphologically, *Sp. prunicola* possesses significantly smaller conidia than those of *Sp. incanus* (12–15 × 3.5–4.5 μm vs. 11.5–20 × 4.5–6.5 μm). Furthermore, *Sp. prunicola* can be clearly distinguished from *Sp. incanus* by 1/515 bp in ITS, 1/830 bp in LSU, 25/832 bp in *rpb2*, 29/289 bp in *tef1*, and 45/712 bp in *tub2*. It also differs from *Sp. italicum* by 2/534 bp in ITS and 4/881 bp in LSU (Hyde et al. 2017; Liu et al. 2019). PHI analysis further supports the status of *Sp. prunicola* as a distinct species, showing no evidence of recombination with its closest relatives (*P* = 1.0) (Fig. 2).

**Figure 3.**
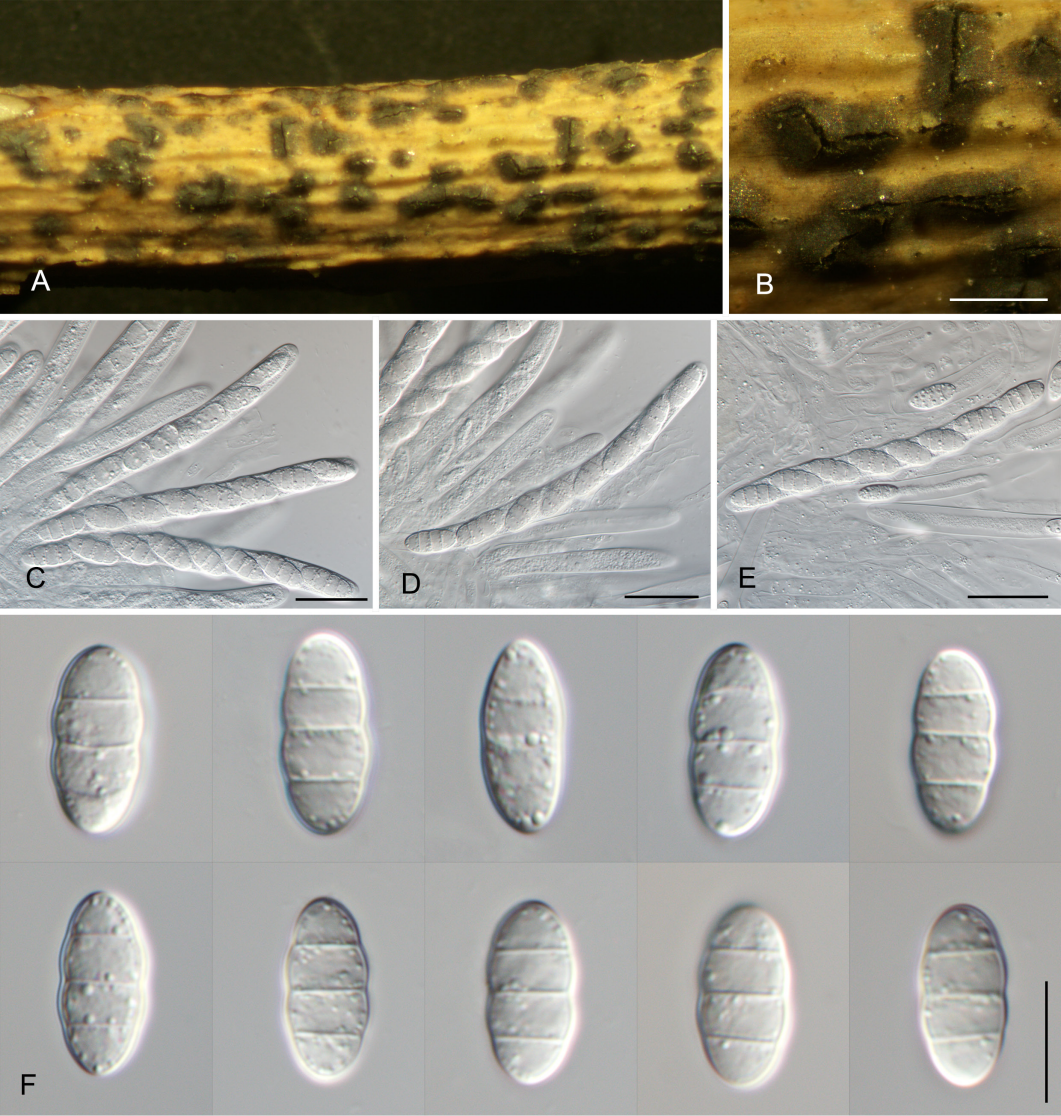
Morphology of *Sporocadus bomiensis*. **A, B**. Appearance of ascomata on host substrate; **C–E**. Asci; **F**. Ascospores. Scale bars: 300 µm (**B**); 20 µm (**C–E**); 10 µm (**F**).

**Figure 4.**
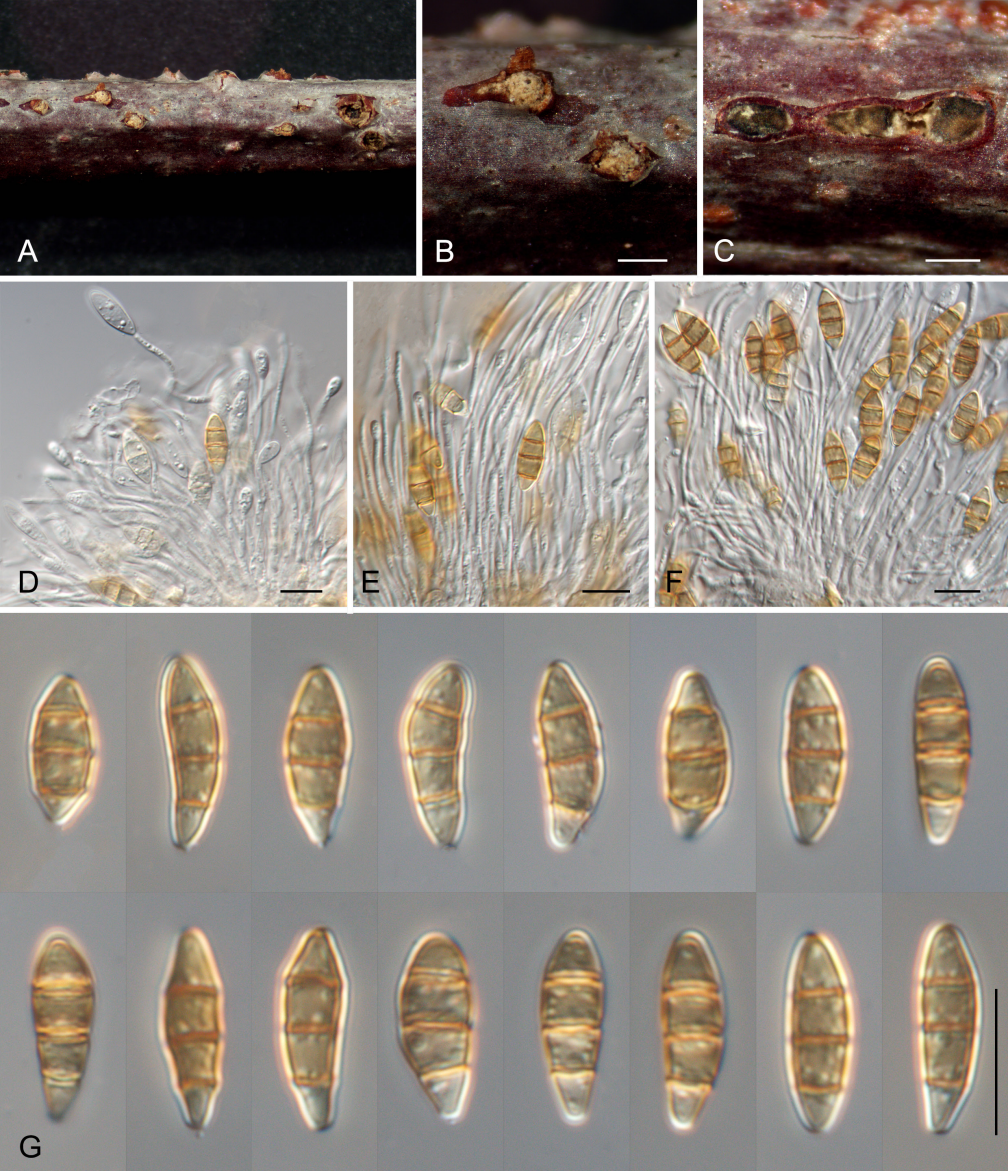
Morphology of *Sporocadus prunicola*. **A–C**. Appearance of conidiomata on host substrate; **D–F**. Conidiogenous cells with attached conidia; **G**. Conidia. Scale bars: 500 µm (**B, C**); 10 µm (**D–G**).

## Discussion

The generic delimitation between *Sporocadus* and *Seimatosporium* has long been one of the most contentious issues within the *Sporocadaceae* (Senanayake et al. 2015; Rossman et al. 2016; Jiang et al. 2019; Liu et al. 2019; Peng et al. 2022). Historically, the presence or absence of conidial appendages was the primary morphological criterion for separating these two genera (Corda 1839; Hughes 1958; Sutton 1975; Nag Raj 1993). However, as demonstrated in our study and several recent phylogenetic revisions, this character is highly plastic and can be homoplastic within the family (Liu et al. 2019). For instance, our new combination, *Sp. parvus* (formerly *Seimatosporium parvum*), possesses distinct appendages but is phylogenetically nested deep within the *Sporocadus* clade with robust support (MLBS = 100%, BIPP = 1.0) (Fig. 1). This finding further supports the hypothesis by Liu et al. (2019) that conidial appendages may have been lost or gained multiple times throughout the evolutionary history of the *Sporocadaceae*.

Our results also reaffirm that ribosomal DNA (ITS and LSU) lacks sufficient resolution to resolve the intergeneric relationships in this complex (Jaklitsch et al. 2016; Liu et al. 2019; Jiang et al. 2022; Peng et al. 2022; Razaghi et al. 2024). The integration of protein-coding genes, particularly *rpb2*, *tef1*, and *tub2*, provided the necessary phylogenetic signal to clarify the natural classification of our collection from Xizang. By employing a five-locus dataset, we were able to define *Sp. bomiensis* and *Sp. prunicola* as distinct lineages, clearly separated from their closest relatives like *Sp. tibetensis* and *Sp. incanus* (Fig. 1). The high statistical support for these clades indicates that a multi-locus approach is essential for accurate species identification and taxonomic stability in pestalotioid fungi.

In addition to the multi-locus phylogeny, the PHI analysis provided a robust statistical framework for species delineation in this study (Fig. 2). For closely related species complexes, such as the *Sp. bomiensis*–*Sp. tibetensis*–*Sp. hyperici* group, simple sequence comparison may sometimes be misleading due to low interspecific variation. However, the PHI test results for both Clade A and Clade B yielded *P*-values of 1.0, indicating a complete absence of significant genetic recombination between the proposed new species and their sister taxa (Fig. 2). This evidence confirms that *Sp. bomiensis* and *Sp. prunicola* are evolving as independent evolutionary lineages.

Xizang is a region of immense biological interest due to its extreme high-altitude environment and unique flora. Our discovery of two new species from rosaceous hosts, *Prunus cerasifera* and *Rosa chinensis*, suggests that the fungal diversity of the Qinghai–Xizang Plateau remains vastly underestimated. Rosaceous plants are well-known hosts for a variety of *Sporocadaceae* members, and the specialized environment of Xizang likely facilitates the diversification of these fungi.

Interestingly, *Sp. bomiensis* was discovered in its teleomorphic state, whereas its closest relatives, *Sp. tibetensis* and *Sp. hyperici*, are currently known only as anamorphs (Liu et al. 2019; Razaghi et al. 2024). This highlights the importance of collecting and analyzing specimens from remote regions to complete the life cycle records of these fungi. Furthermore, the association of *Sp. prunicola* with branch cankers on *Prunus cerasifera* indicates that this genus may play a significant role in plant health within alpine ecosystems, either as primary or secondary pathogens.

The transfer of *Seimatosporium parvum* to *Sporocadus* as *Sp. parvus* represents a necessary taxonomic revision based on our five-gene phylogeny (Liu et al. 2019; Peng et al. 2022). Although this species morphologically “fits” the traditional concept of *Seimatosporium* due to its appendages, its genetic core is unmistakably *Sporocadus* (Corda 1839; Hughes 1958; Sutton 1975; Nag Raj 1993). This taxonomic change emphasizes the guiding principle in modern fungal systematics: when morphological traits conflict with robust multi-locus phylogenetic data, the latter should be prioritized to reflect natural evolutionary relationships.

In conclusion, this study provides new insights into the diversity and systematics of *Sporocadus* in China. By combining morphology, a five-locus phylogeny, and PHI analysis, we have successfully characterized two new species and proposed a new combination. Our work underscores the necessity of moving beyond single-locus DNA barcoding and traditional morphological criteria for the classification of pestalotioid fungi. Future research should continue to explore the fungal wealth of the Xizang Plateau, which likely holds more keys to understanding the evolutionary trajectory of the *Sporocadaceae*.

## Supplementary Material

XML Treatment for Sporocadus bomiensis

XML Treatment for Sporocadus parvus

XML Treatment for Sporocadus prunicola

## Additional information

### Artificial Intelligence (AI) use

The authors accept full responsibility for the content of the manuscript, including the disclosure of any use of AI.

No AI tools were used in the preparation of this manuscript.

### Author ORCIDs

Z. Li https://orcid.org/0009-0006-9715-4170

C. Tian https://orcid.org/0000-0002-3352-7664

N. Jiang https://orcid.org/0000-0002-9656-8500

### Data availability

All of the data that support the findings of this study are available in the main text.
